# Supplementary figures and images for: The genetic architecture of changes in adiposity during adulthood
Source: medRxiv. 2023 Jan 11:2023.01.09.23284364. Preprint. [Version 1] doi: 10.1101/2023.01.09.23284364 (PMC9882550; doi:10.1101/2023.01.09.23284364)

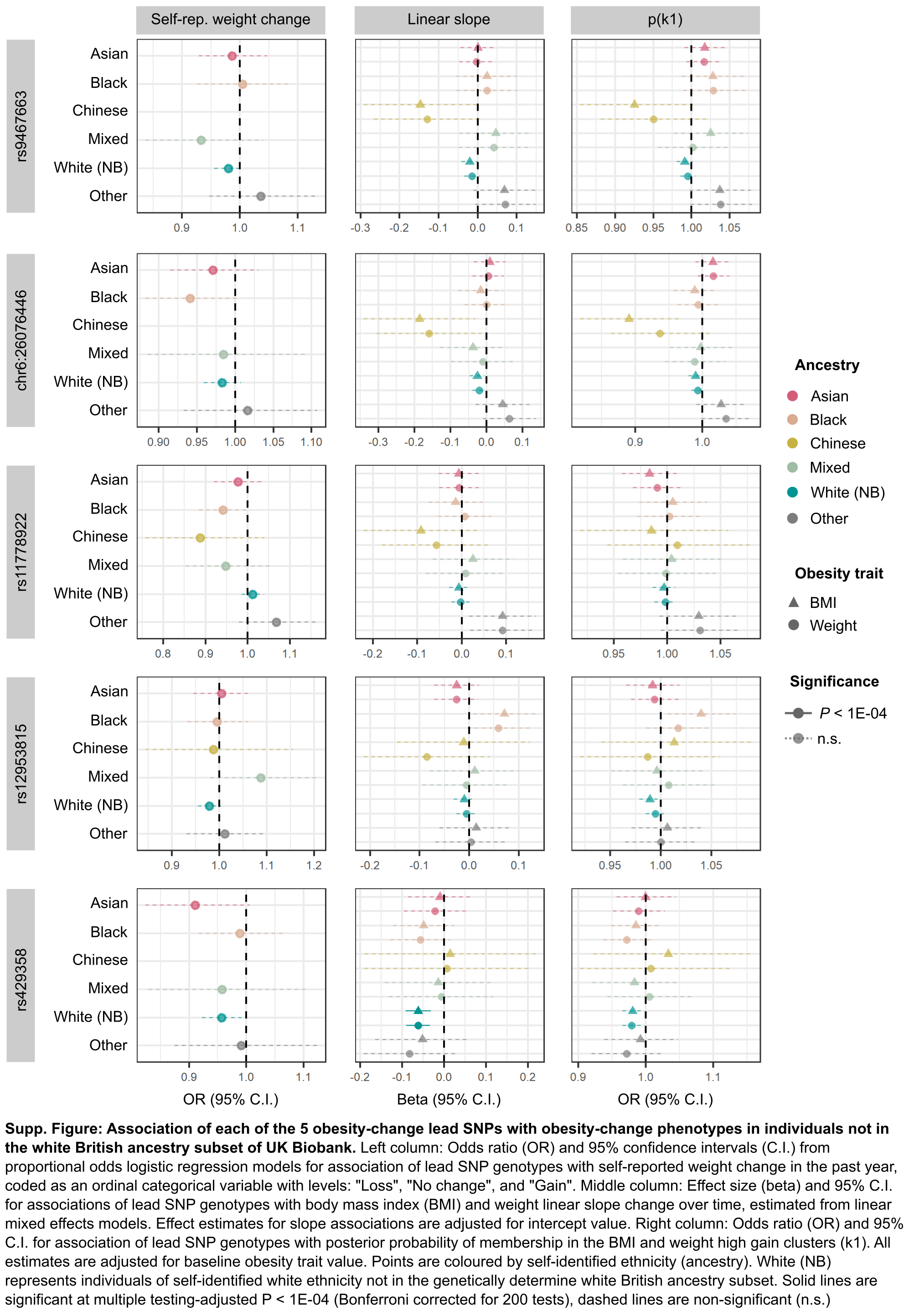

Supplement: Supplement 2 [file media-2.zip › Supp-Fig-1-supp-results-nonwb-ancestry.png]

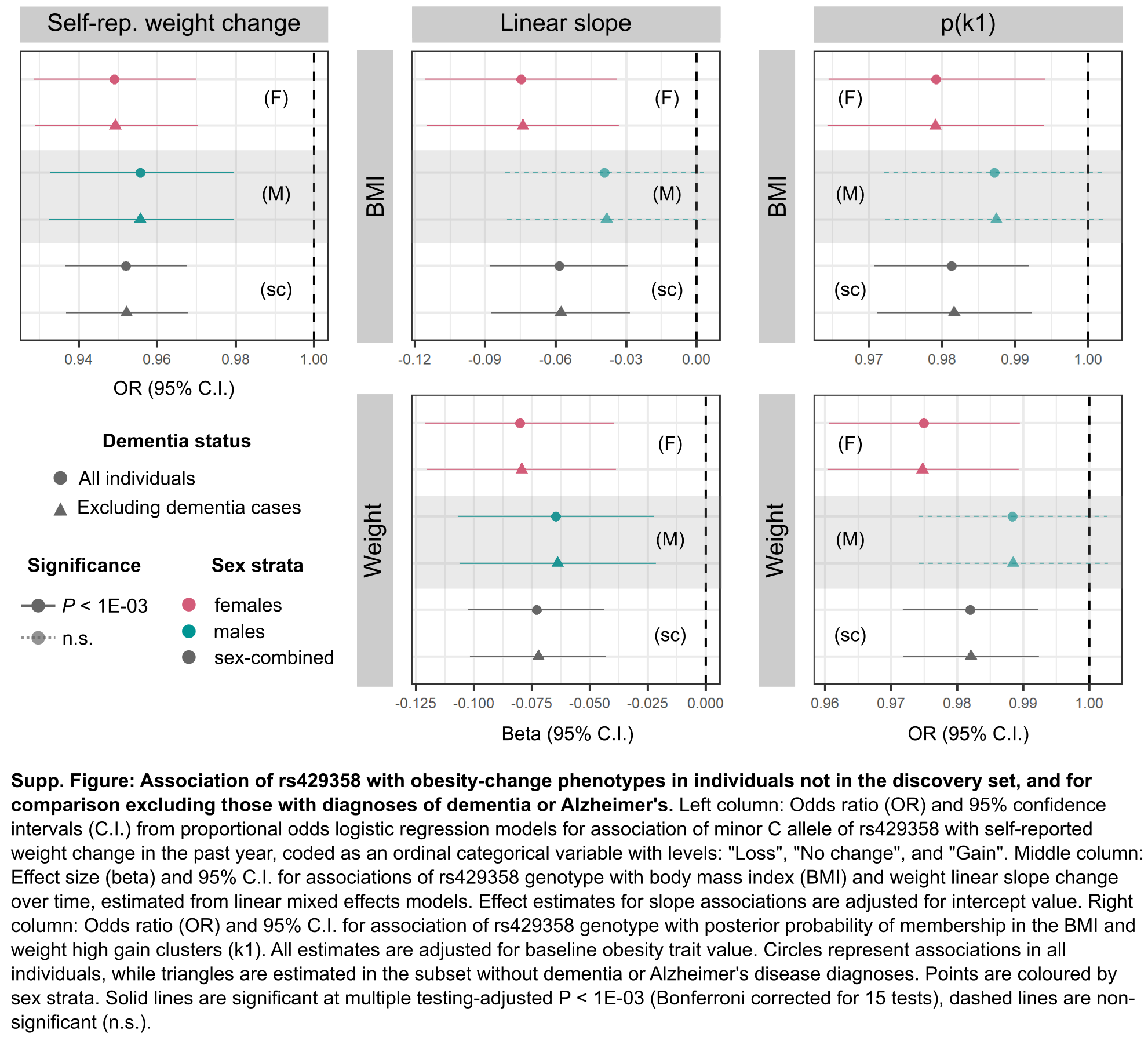

Supplement: Supplement 2 [file media-2.zip › Supp-Fig-2-supp-results-rs429358-dementia.png]

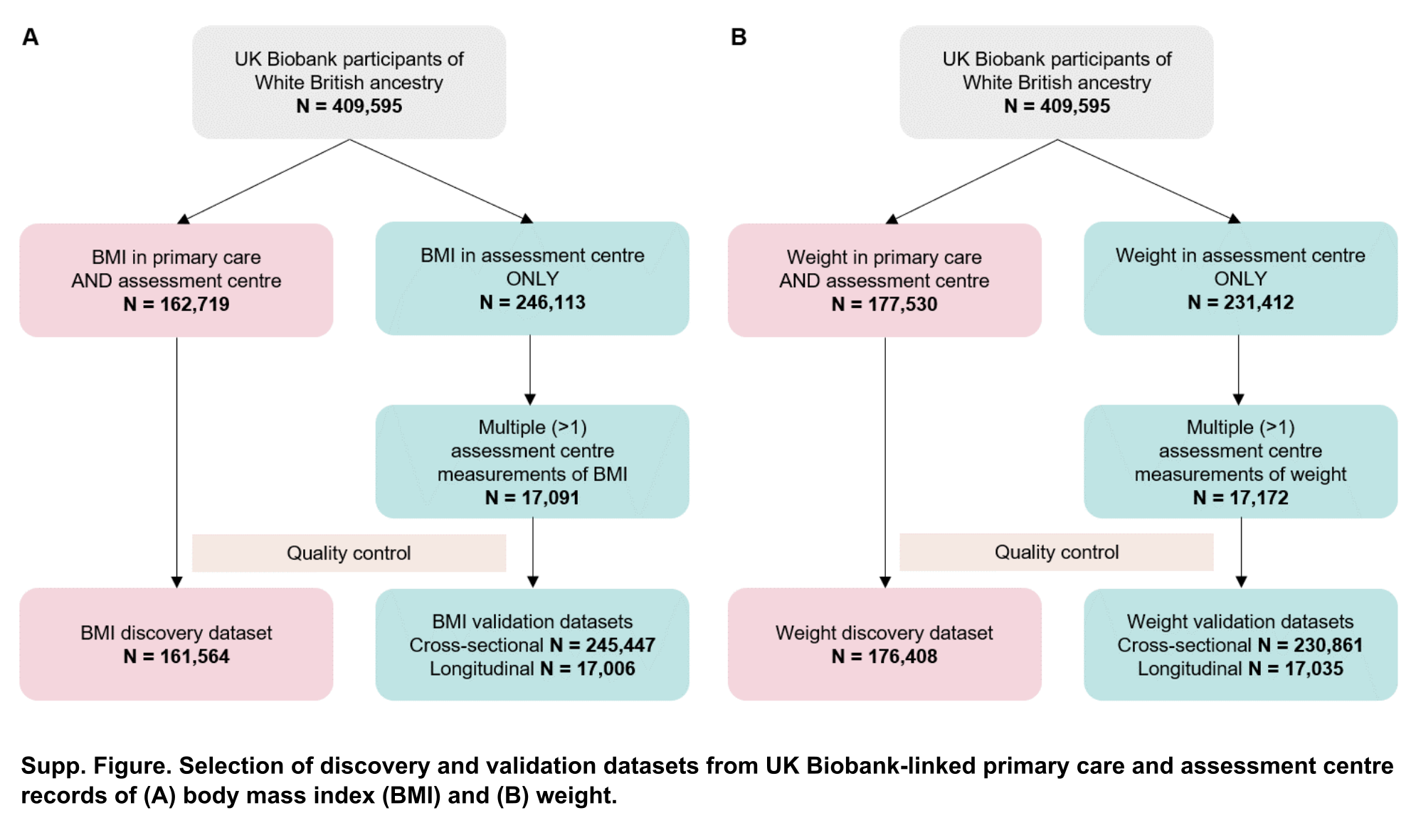

Supplement: Supplement 2 [file media-2.zip › Supp-Fig-3-supp-methods-data.png]

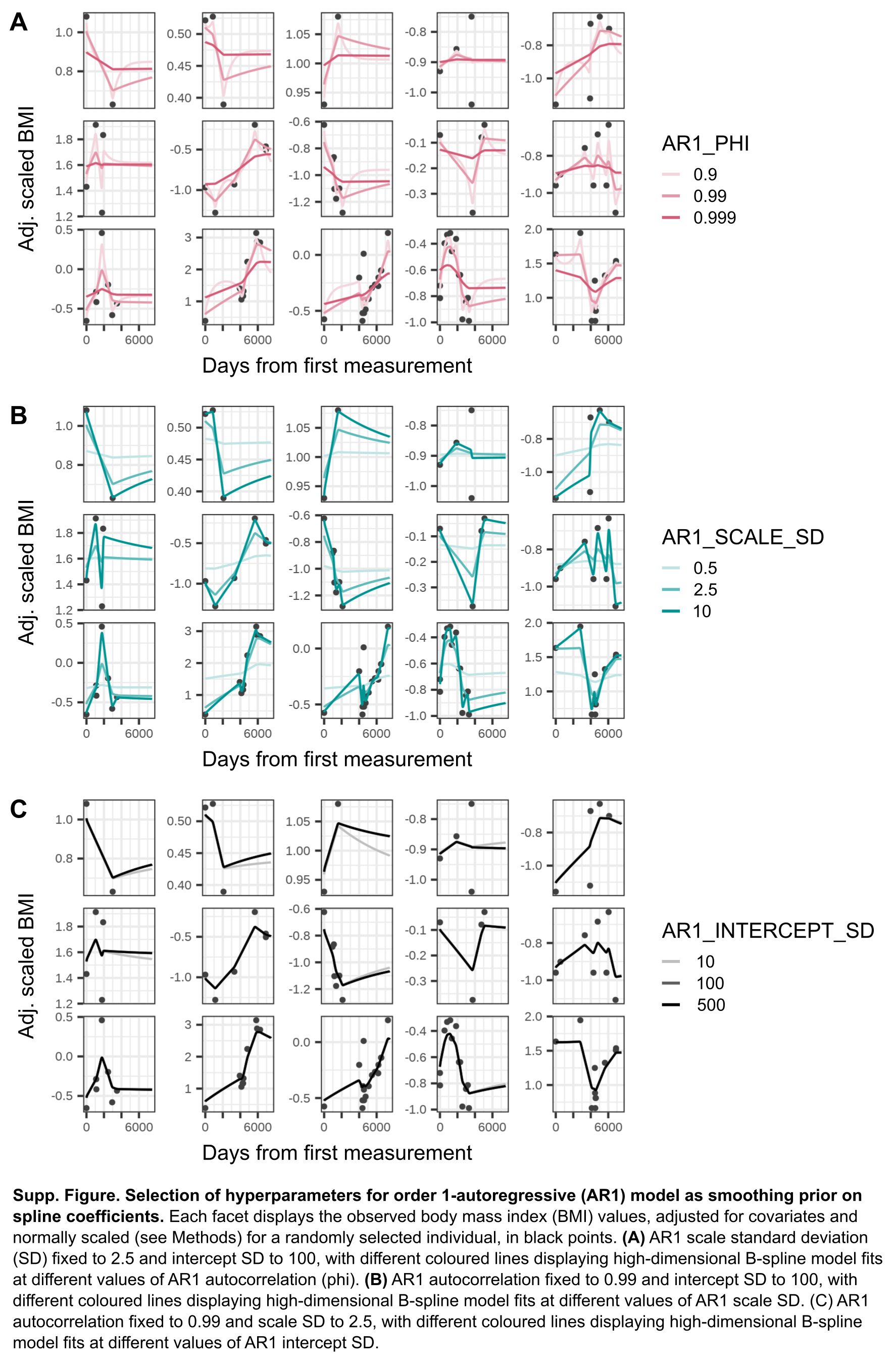

Supplement: Supplement 2 [file media-2.zip › Supp-Fig-4-supp-methods-ar1-hyperparameters.png]

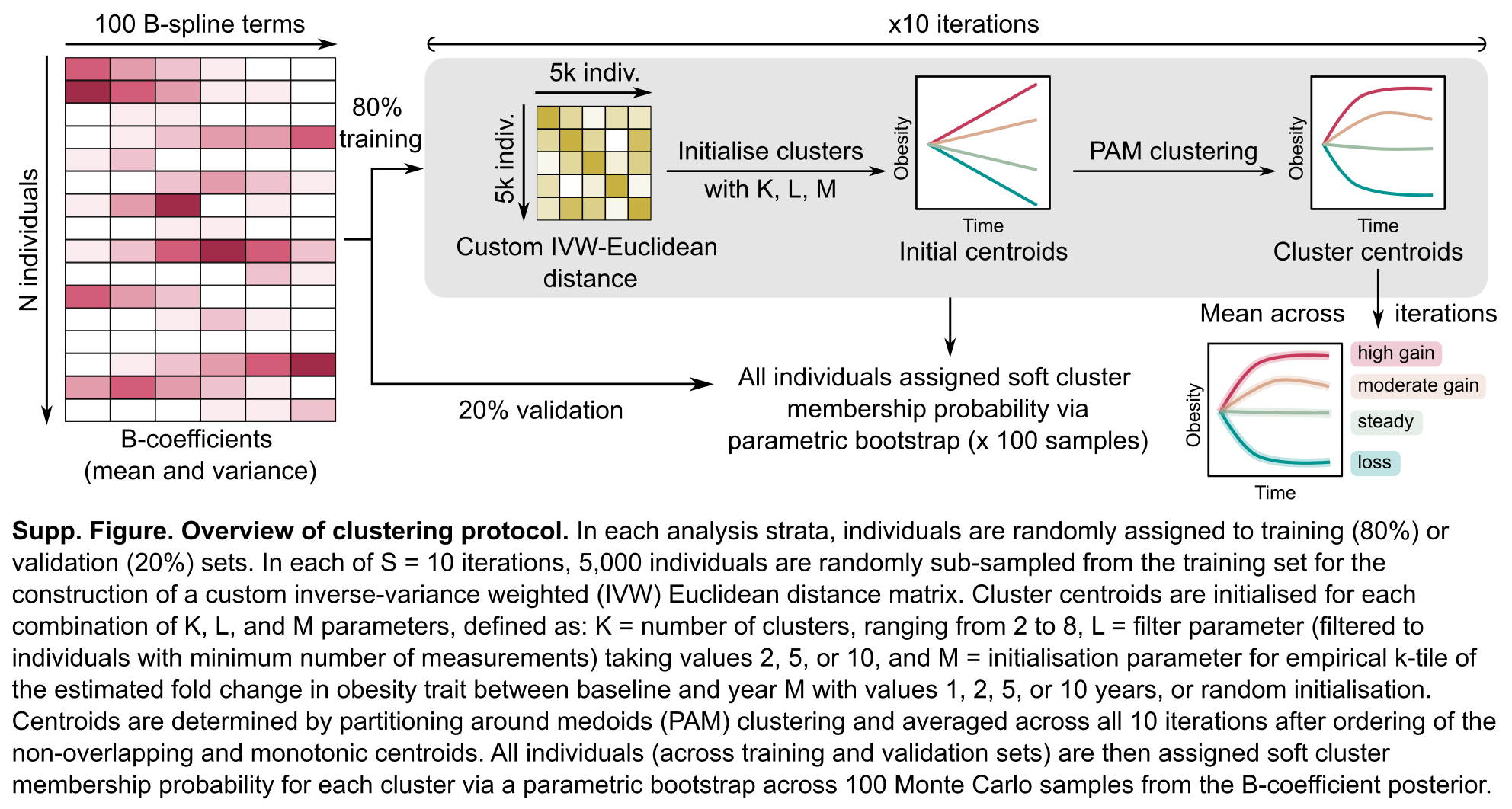

Supplement: Supplement 2 [file media-2.zip › Supp-Fig-5-supp-methods-clustering-overview.png]

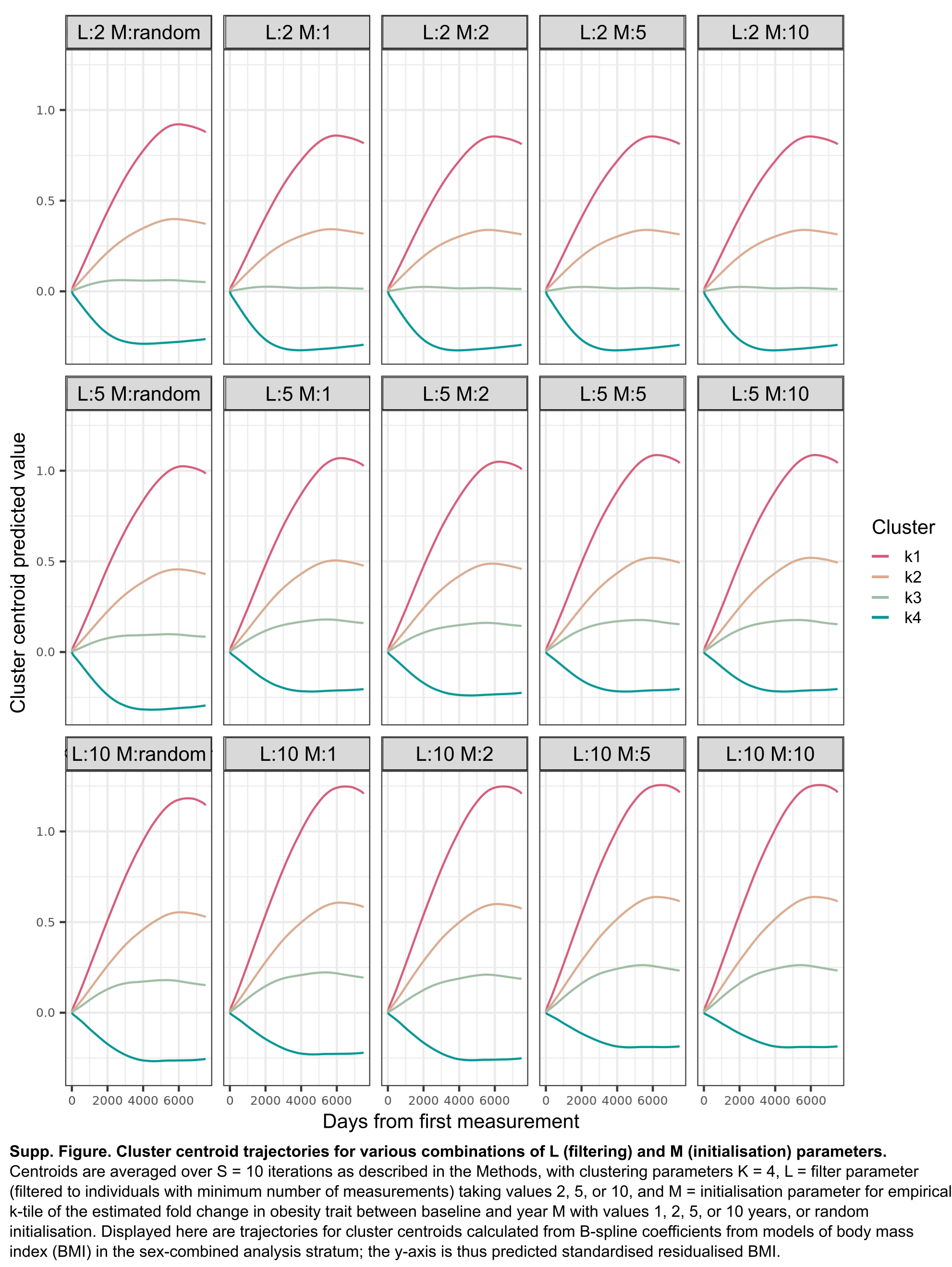

Supplement: Supplement 2 [file media-2.zip › Supp-Fig-6-supp-methods-cluster-centroids.png]

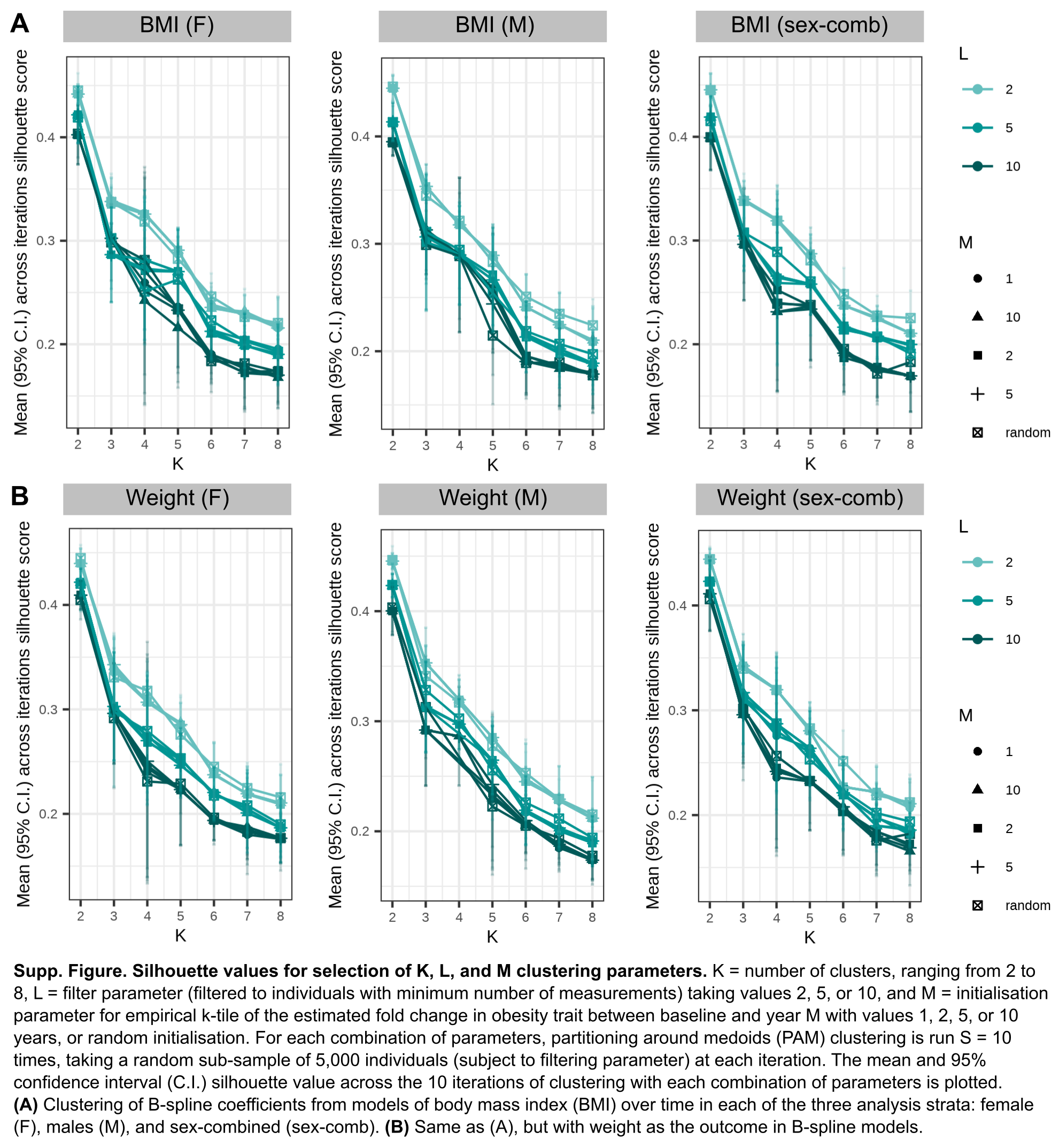

Supplement: Supplement 2 [file media-2.zip › Supp-Fig-7-supp-methods-klm-silhouettes.png]

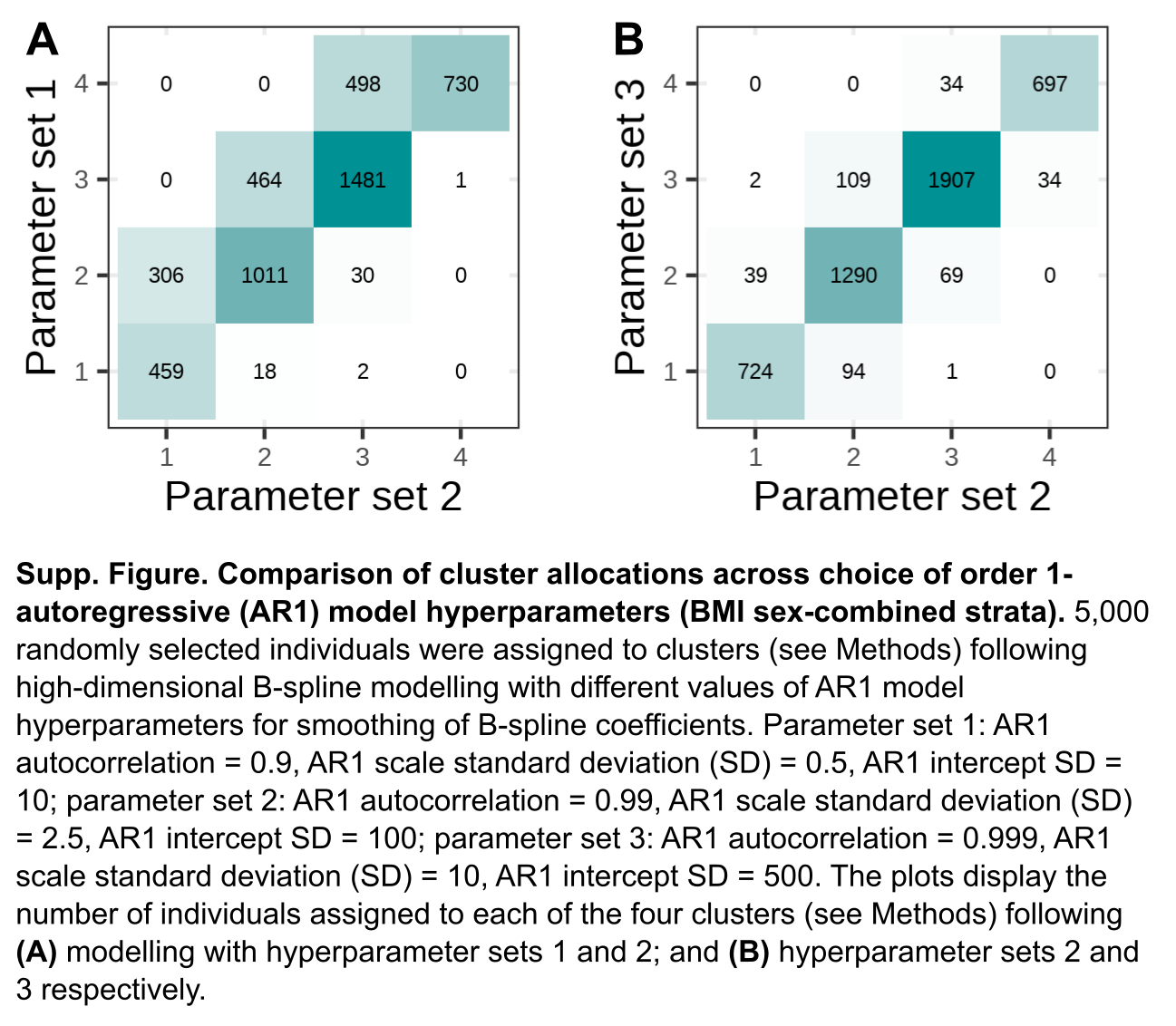

Supplement: Supplement 2 [file media-2.zip › Supp-Fig-8-supp-methods-clustering-sensitivity-ar1-choices.png]

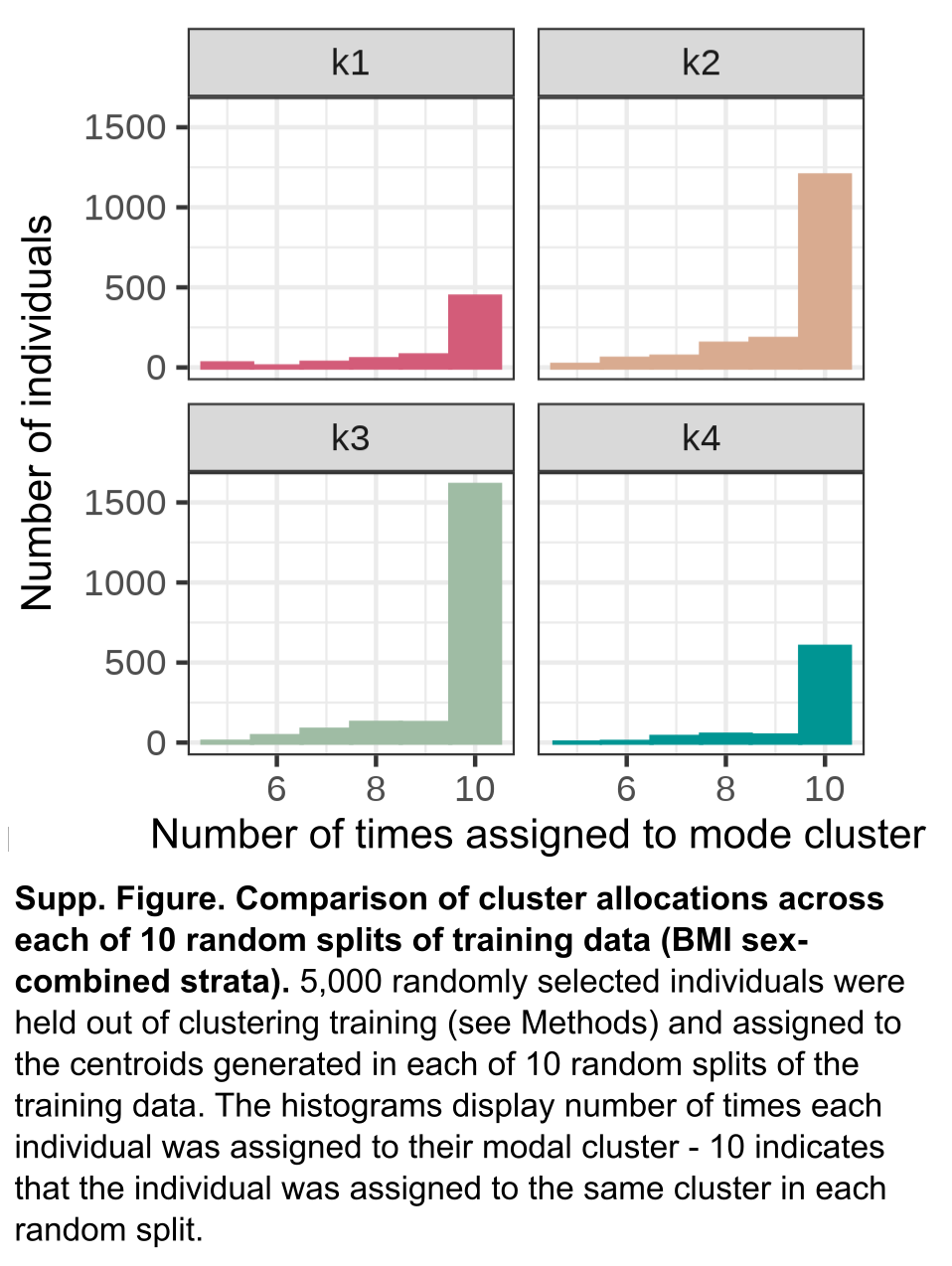

Supplement: Supplement 2 [file media-2.zip › Supp-Fig-9-supp-methods-clustering-sensitivity-random-train.png]
